# Indents

**Published:** 1919-07

**Authors:** 


### INDENTS

☞Brief Articles of Technical or Scientific Value will receive notice and comment. Contributions invited.

---

THE GATEWAY OF SORROWS. "Where would you like to be sent to live in France," asked the clerk at Evian.

The little old woman's eyes slowly filled with tears and her answer moved to pity all who heard it. "If I could only live near a cathedral—I have always lived so close to Rheims." Her sons dead in the war, her sisters killed in the bombardment of their village, the frail little old woman clung to the last familiar landmark of the beloved home, and so under the shadow of beautiful Chartres Cathedral this lonely French woman will end her days.

In recording the many splendid things the American Red Cross did for the civilian population in France during the fourth winter of the war one can not but pause to pay tribute to the magnificent work done by the French government, in which we have been privileged to assist.

Among the many beautiful things, touched with the spiritual sentiment of the French, none makes a stronger appeal than the story of the little children who pass through that "gateway of a hundred sorrows."

Hundreds of them, homeless, countryless, fatherless, motherless, orphans. On their pitiful garments were sewed the ugly tags of identification. How that crude recording has hurt the French is shown in their quick substitution of the pretty bone chains. If you had only a mother you wore a blue one, the blue of France; if just a father you wore a red one, and if you had no one the little white chain was yours. And no one but the officials knew the significance of these chains.

Little things, just little tender things those—but they have helped these people bear the long weary burdens of the war.

DENTISTRY AND CANCER. Cancer of the mouth in civilized countries has been greatly reduced by good dentistry. Eighty-five per cent. of the cancers of the lip occur in smokers. Formerly clay pipes, which became very hot, were much used, and there has been a notable reduction in the number of cancers of the lip since the clay pipe has gone out of fashion. Smoking, however, is the cause of most cancer of the lip, the tongue and the floor of the mouth.—Major W. J. Mayo, War Department Lectures.

---

TO PREVENT EXPANSION OF PLASTER IN SETTING. If slacked lime is added to boiling water, allowed to cool, and the clear liquid decanted for use in mixing the plaster of paris, the plaster will not expand.—*International Dental Journal.*

---

TRENCH MOUTH. Professor Adami was informed by the Director of the Canadian Dental Service that at Whiteley Camp there was a striking reduction in the cases of "trench mouth" after an order was issued that the rim of every glass or mug used in the canteen should be dipped in boiling water in the presence of the customer. The order was also enforced at all inns and bars of the neighborhood. All drinking places that had not installed proper facilities for doing this were put out of bounds. Food utensils were also sterilized. Similar precautions should be taken in every public drinking place.—*Dental Record.*

---

TEETHING. Suñer reiterates that the teething period in infants is not pathologic but is a period of stress and adjustment, like puberty and the menopause. Inherited taints may first make themselves manifest at such periods, and various pathologic conditions otherwise may prove refractory to all treatment until the stress of this critical epoch is somewhat mitigated.—*Journal A. M. A.*, June 21, p. 1877.

Securing Canal Broaches. To prevent dropping short canal broaches into the throat always apply the rubber dam before using, covering enough teeth to furnish ample space for manipulating the broach. If, for any reason, the dam can not be adjusted, tie a waxed ligature to the hub and leave enough ligature to extend well out of the mouth. This will allow of all proper manipulation and render it possible to rescue the broach in case it slips out of the finger grasp and prevent its dropping into the fauces. A piece of orthodontia wire can also be attached to the hub of the broach, but it does not give as much freedom in manipulation.

---

Theory of Asthma. The underlying cause of asthma, according to Knapp, is the overproduction of irritating gases in the gastro-intestinal canal, which, operating physically and chemically, produce the asthmatic paroxysm. 1. Physically, by overdistention of abdomen and of the pressure upward of the diaphragm against the lungs, whose expansion is violently interfered with. 2. Chemically, by irritating the esophagus and pharynx, which irritation the patient attempts to clear away by the act of coughing. So long as the irritation continues, so long continues the ineffective coughing. The longer the irritation continues, the more violent becomes the act of coughing, the consequence of which is dilatation of the pulmonary air vesicles, which may and often does terminate in emphysema of the lungs. The asthmatic paroxysm ends when the irritation in the esophagus and pharynx have been removed, partly by neutralization of the irritation and partly by its dilution with pharyngo-esophageal secretions.—*Journal A. M. A.*, March 22.

---

Casting Inlays. The existing differences of opinion as to the right method and technique of casting gold inlays would probably vanish and be heard no more if only there was *one* method by which every operator could in actual fact and without fail produce a perfectly fitting inlay every time. Puzzling failure or partial failures do sometimes occur, however, and explanations are generally interesting. Mr. F. W. Tompson says in the *Commonwealth Dental Review* that small inlays were often a shade too large when cast in a *hot* mold, while large inlays were too small for the cavity when cast in a cold mold. His explanation of this seeming anomaly is as follows:

"With large inlays more heat can be applied to the investment, bringing it to a dull-red color with good results. The explanation seems to be that the investment material, if brought to a very high heat, will shrink very considerably, thereby causing the inlay to be larger than the wax model by the amount of the shrinkage of investment minus the shrinkage of the gold as it cools, which, in the case of small inlays, is considerably less than the shrinkage of the investment, while with the larger mass of gold in the case of large inlays, the shrinkage of the gold comes nearer to compensating for the shrinkage of the investment. I find that if large inlays are cast into a very cool investment the inlay will come out too small for the cavity, the margins showing a decided shrinking away. Also, I find that better results can be obtained by using as small an investment as possible."—*Dental Record.*

---

REMOVING PLASTER FROM VULCANITE DENTURES. The dental laboratory worker sometimes finds, on removing a plate from the flask after vulcanization, that the mold or model plaster has formed a hard and strongly adherent layer of crystals on the surface of the vulcanite. Prevention is, of course, better than cure, and one ought not to be so careless as to leave the flask in water for some hours after vulcanization. In cases where one can afford to wait for the gradual action of a slow solvent, the following method will be found very satisfactory: The well-washed plate should be immersed in a strong or saturated solution of sodium hyposulphite—photographers' "hypo"

—and left in this solution overnight. On removal from the solution, it will be found that, in many instances the incrustation has been entirely dissolved. Should some crystals remain on the plate, they no longer adhere closely, and they can be readily brushed away, leaving the vulcanite surface quite clean.—*Oral Health.*

---

The Harvard Surgical Unit. Early in 1915 Harvard sent to England a full hospital unit fitted out in all its branches. At the head of its dental section was Dr. K. I. Kazanjian, the University Demonstrator in Prosthetic Dentistry. Throughout the weary years of war Dr. Kazanjian and his colleagues, deserting their home practice, stuck to their noble work. The university has loyally supported them throughout. On one occasion it became accidentally known that the head of the dental section had spent £200 from his own pocket upon equipment which was needed for his work. At a meeting of the Harvard Alumni Society the matter was mentioned, and so eager was every graduate present to assist that the amount was then and there oversubscribed. On Saturday, March 15, at Buckingham Palace, His Majesty the King, invested Major Kazanjian with the Insignia of a Companion of the Order of St. Michael and St. George. The members of the Harvard Surgical Unit have now sailed for America. They carry with them our grateful thanks. Promptly at the outbreak of war they came to our aid. While they have been in France, their knowledge and skill has not only been at the disposal of our wounded, but have been made fully available for those of us who were engaged in similar work here. This mission has forged a new link between Harvard and the dental schools of this country, which will be gratefully remembered long after the echoes of war have died away.—*British Dental Journal.*

